# Sublingual allergen immunotherapy: evidence from randomized trials, real-world studies and meta-analyses

**DOI:** 10.3389/falgy.2026.1940709

**Published:** 2026-09-14

**Authors:** Giovanni Paoletti, Nicolò Valli, Danilo Di Bona, Giulia Anna Maria Luigia Costanzo, Andrea Giovanni Ledda, Stefano Del Giacco, Mattia Giovannini, Francesco Catamerò, Francesca Mori, Giuseppe Guida, Fabio Luigi Massimo Ricciardolo, Francesca Bertolini, Remo Poto, Corrado Pelaia, Gilda Varricchi, Giorgio Walter Canonica, Enrico Heffler

**Affiliations:** 1 Department of Biomedical Sciences, Humanitas University, Pieve Emanuele, Italy; 2 Personalized Medicine, Asthma and Allergy, IRCCS Humanitas Research Hospital, Rozzano, Italy; 3 Department of Medical and Surgical Sciences, University of Foggia, Foggia, Italy; 4 Department Medical Science and Public Health, University of Cagliari, Cagliari, Italy; 5 Department of Health Sciences, University of Florence, Florence, Italy; 6 Allergy Unit, Meyer Children's Hospital IRCCS, Florence, Italy; 7 Department of Clinical and Biological Sciences, University of Turin, Turin, Italy; 8 Severe Asthma, Rare Lung Disease and Respiratory Pathophysiology Unit, San Luigi Gonzaga University Hospital, Orbassano (TO), Italy; 9 Institute of Translational Pharmacology, National Research Council (IFT-CNR), Palermo, Italy; 10 Department of Translational Medical Sciences, University of Naples Federico II, Naples, Italy; 11 Center for Basic and Clinical Immunology Research (CISI), University of Naples Federico II, Naples, Italy; 12 Department of Medical and Surgical Sciences, University “Magna Graecia” of Catanzaro, Catanzaro, Italy

**Keywords:** allergen immunotherapy, allergic asthma, allergic rhinitis, clinical remission, disease modification, house dust mite, pollen allergy, sublingual immunotherapy

## Abstract

**Background:**

Allergen immunotherapy is the only potentially disease-modifying treatment for IgE-mediated respiratory allergy. Among the available routes, sublingual immunotherapy (SLIT) has gained prominence because of its favorable safety profile and greater patient acceptability.

**Objectives:**

This narrative review critically appraises the clinical efficacy, real-world effectiveness, and long-term disease-modifying potential of SLIT for seasonal and perennial respiratory allergies by integrating evidence from randomized controlled trials, large observational cohorts, and meta-analyses, with attention to allergen- and product-specific outcomes and age-related differences.

**Results:**

Robust RCT evidence supports clinically meaningful reductions in symptoms and medication use with grass and HDM SLIT tablets, with sustained benefits observed up to 1–2 years after a 3-year treatment course in key trials. Real-world cohorts corroborate these findings and additionally report a reduced risk of new asthma onset and lower long-term medication dispensing after SLIT. Evidence for SLIT in asthma shows reductions in exacerbation risk in some trials, although its effects on day-to-day control are less consistent. Pediatric trials generally mirror adult outcomes, with favorable safety and efficacy, particularly for standardized tablets; however, heterogeneity across trials and historical methodological limitations remain important caveats. Meta-analyses confirm overall efficacy but highlight substantial between-study heterogeneity and the importance of product-specific evaluations.

**Conclusions:**

SLIT could be an effective, well-tolerated, disease-modifying option for respiratory allergic rhinitis and shows promise for selected asthma outcomes. Optimal benefits require standardized, adequately dosed formulations and sustained adherence (≥3 years). Future research should prioritize product-specific, adequately powered trials with standardized outcomes to refine patient selection and long-term disease-modification endpoints.

## Introduction

Allergic rhinitis and asthma are among the most prevalent chronic allergic disorders worldwide and continue to impose a substantial burden on both children and adults (1). Beyond their epidemiological relevance, these conditions significantly impair quality of life (2), affecting sleep quality (3), daytime functioning, school performance, and work productivity (4). Moreover, allergic rhinitis frequently coexists with asthma, supporting the concept of a common inflammatory airway disease (1, 5). Although environmental control and symptomatic pharmacotherapy remain essential components of management, neither strategy can modify the underlying immunopathology or prevent long-term disease progression (1). In this context, allergen immunotherapy (AIT) remains the only intervention capable of inducing disease-modifying effects in IgE-mediated respiratory allergies (6). More recently, AIT has been framed within a remission-oriented therapeutic paradigm, although standardized definitions and prospectively validated remission endpoints remain to be established (7).

In this evolving therapeutic landscape, the 2026 World Allergy Organization (WAO) position paper provides an updated, internationally harmonized, evidence-informed framework for the use of SLIT, integrating data on efficacy, safety, treatment adherence, disease modification, and implementation across diverse healthcare settings (8).

AIT can be delivered via subcutaneous immunotherapy (SCIT) or sublingual immunotherapy (SLIT). Both routes have demonstrated efficacy in reducing symptoms and medication use in allergic rhinoconjunctivitis, with additional benefits in selected patients with allergic asthma (9, 10). However, the two approaches differ substantially in terms of administration, safety, and patient acceptability (10, 11). SCIT requires repeated injections under medical supervision, usually with post-administration observation, whereas SLIT is initiated under supervision but subsequently continued by the patient at home through daily self-administration (11). These practical differences are clinically relevant, as adherence and long-term persistence are key determinants of effectiveness, particularly given that at least three years of continuous treatment are generally required to obtain sustained post-treatment benefits (12).

Among the available AIT strategies, SLIT has progressively gained prominence because it combines a favorable efficacy profile with greater convenience and an improved safety profile compared with that of SCIT (1). In routine practice, this advantage is particularly relevant for patients who are reluctant to undergo injections or have limited access to specialist centers (1). The rationale for focusing on SLIT lies not only in its established clinical efficacy but also in its lower burden of treatment delivery and predominantly local, self-limited adverse event profile.

The therapeutic effect of SLIT is based on the induction of allergen-specific immune tolerance rather than simple transient symptom suppression (13). Following sublingual administration, the allergen is taken up by antigen-presenting cells within the oral mucosa, especially dendritic cells with tolerogenic properties, which process and present the allergen in a manner that favors immune regulation instead of type 2 inflammatory amplification (14). This early interaction at the oral mucosa level is considered a pivotal event in the mechanism of SLIT because it initiates a cascade of regulatory signals that can reshape both innate and adaptive immune responses (13). Over time, these changes translate into reduced clinical sensitivity to natural allergen exposure and a more stable state of immune tolerance (1, 13).

At the cellular level, SLIT promotes a shift away from Th2-dominated immunity toward a more regulated immune phenotype (13). Regulatory T cells, including inducible Treg subsets, play a central role in producing interleukin-10 and transforming growth factor-beta and suppressing allergen-specific effector responses (14). These mediators inhibit T cell proliferation, dampen eosinophilic and mast-cell–driven inflammation, and contribute to the development of peripheral tolerance (13). In parallel, increasing evidence suggests that regulatory B-cell responses and additional mucosal immune pathways participate in maintaining this tolerant state, further supporting the concept that SLIT acts through coordinated immune reprogramming rather than a single dominant mechanism (15).

Humoral changes are also a well-recognized component of SLIT-induced immune modulation (13). During treatment, allergen-specific IgE may initially increase and subsequently decline, whereas allergen-specific IgG4 levels rise and act as blocking antibodies capable of interfering with IgE-facilitated allergen recognition (16). This mechanism limits allergen-induced cross-linking of IgE on mast cells and basophils, thereby reducing effector cell activation. Notably, compared with SCIT, SLIT appears to induce a more pronounced mucosal IgA response, a finding that is biologically coherent with its route of administration and may be relevant for local immune protection at the respiratory mucosal surfaces (17). Altogether, the immunological basis of SLIT can be understood as the integrated result of tolerogenic dendritic cell signaling, expansion of regulatory lymphocyte populations, suppression of Th2 inflammation, and production of non-inflammatory blocking antibodies (18).

Against this background, this narrative review critically appraises the efficacy and real-world effectiveness of SLIT in respiratory allergies, integrating evidence from randomized controlled trials, observational studies, and meta-analyses, with particular attention to allergen- and product-specific outcomes, age-related differences, and long-term effects.

## Methods

This manuscript was prepared as a non-systematic narrative review aimed at critically synthesizing the current evidence on SLIT for respiratory allergic diseases. A literature search was primarily performed using PubMed/MEDLINE, with the last search conducted in March 2026. The search terms included combinations of “*sublingual immunotherapy”*, “*SLIT”*, “*allergic rhinitis”*, “*allergic asthma”*, “*house dust mite”*, “*grass pollen”*, “*randomized controlled trial”*, “*real-world study”*, “*systematic review*” and “*meta-analysis”*. Preference was given to more recent and clinically significant studies, although no predefined publication date limits were applied to the search. Evidence was selected based on clinical relevance and methodological quality, with an adequately sized population. When studies evaluated allergen immunotherapy more broadly, only data specifically referring to patients treated with SLIT were considered.

## Evidence from randomized controlled trials and real-world studies

### SLIT in allergic rhinitis

In seasonal allergic rhinitis, the efficacy of SLIT is supported by the most robust evidence (Figure 1). In a landmark phase III randomized, double-blind, placebo-controlled trial, Durham et al. (19) demonstrated that three years of treatment with a standardized grass SLIT-tablet resulted in sustained clinical efficacy for up to two years after treatment discontinuation. Compared with placebo, SLIT significantly reduced symptoms and medication scores across five consecutive pollen seasons, with sustained improvements in quality of life *(*QoL) and fewer days with severe symptoms compared with placebo. Importantly, these long-term clinical benefits were paralleled by persistent immunological changes, including increased allergen-specific IgG4 and IgE blocking activity.

**Figure 1:**
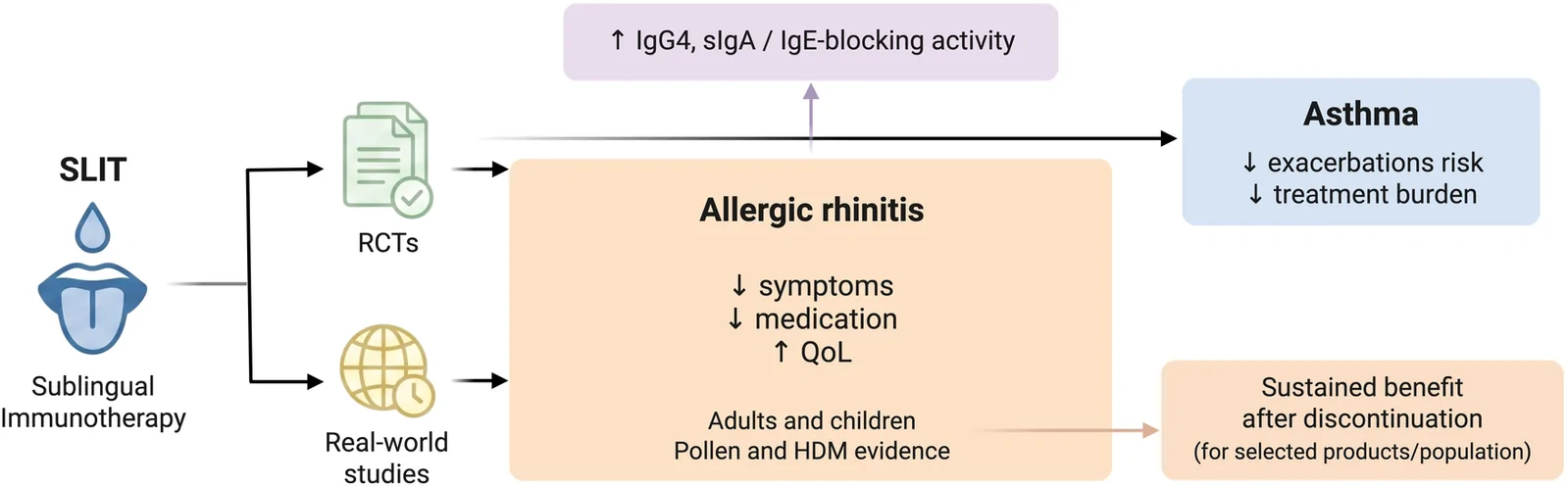
Schematic overview of the effects of SLIT on allergic disease. Evidence from randomized controlled trials and real-world studies indicates reduced symptoms and medication use in allergic rhinitis, improved quality of life, and increased IgG4, secretory IgA (sIgA)/IgE-blocking activity. In asthma, SLIT is associated with fewer exacerbations and reduced use of asthma medications. Sustained clinical benefits after treatment discontinuation have been demonstrated in both adults and children, with evidence reported for pollen and house dust mite allergens. Created with BioRender.com.

Although Randomized controlled trials (RCTs) remain the gold standard, evidence from real-world studies further supports the long-term effectiveness of SLIT in routine clinical practice. In a large retrospective study (20) including 1,099 patients treated with grass pollen SLIT tablets and over 27,000 controls, SLIT was associated with a marked reduction in symptomatic medication use, with approximately half of the treated patients showing sustained reductions in treatment burden and more than one-third requiring no symptomatic medication after treatment discontinuation. In addition, SLIT was associated with a 63.7% lower risk of new asthma onset after age matching (95% CI: 31.5%–80.7%) and reduced asthma medication use during follow-up.

Similar findings were reported by Zielen et al. (21). In a large population-based cohort study including 2,851 SLIT-treated patients and more than 71,000 controls, SLIT was associated with sustained reductions in allergic rhinitis medication use after treatment cessation, delayed asthma onset, and lower asthma medication requirements over time. These findings were further reinforced by the nationwide EfficAPSI real-world study (22), including more than 445,000 patients, in which liquid SLIT was associated with a lower risk of asthma onset and slower asthma progression, further supporting the disease-modifying potential of AIT.

Further support was derived from a large German real-world cohort analysis (23) evaluating multiple AIT formulations, including both SLIT and SCIT, for birch pollen-associated allergic rhinitis. In this study, AIT-treated patients showed significantly lower use of symptomatic rhinitis medication during follow-up than controls, with comparable effectiveness observed across different AIT formulations, including SLIT, further supporting the consistency of treatment effects in seasonal allergic rhinitis under real-world conditions.

Comparable benefits have also been demonstrated in perennial allergic rhinitis, particularly in house dust mite-driven diseases. Evidence for the efficacy of SLIT in perennial allergic rhinitis is supported by both randomized trials and long-term, real-world data.

In a large multicenter, double-blind, placebo-controlled phase III trial (24) including 968 patients with house dust mite (HDM)-induced allergic rhinitis, treatment with HDM SLIT tablets for 52 weeks significantly improved symptom scores, rescue medication use, and QoL compared with placebo, with clinical efficacy observed as early as 8–10 weeks after treatment initiation. Importantly, the treatment was generally well tolerated, with adverse events predominantly mild and local in nature, and no treatment-related anaphylaxis was reported.

These findings were further reinforced by long-term real-world data. In a large Japanese post-marketing surveillance study (25) evaluating 300 IR HDM SLIT tablets over up to four years, sustained and progressive clinical improvement was observed during treatment. The proportion of patients with severe allergic rhinitis symptoms decreased markedly from 46.4% at baseline to 1.0% after four years, whereas the proportion of asymptomatic patients increased to 57.7%. Simultaneously, the use of symptomatic medications decreased during treatment, further supporting a clinically relevant reduction in the disease burden. Notably, clinical benefits persisted for up to one to two years after treatment discontinuation in patients who stopped therapy because of clinical improvement, supporting the potential long-term disease-modifying effect of SLIT.

### SLIT in allergic asthma

While the efficacy of SLIT is well established for allergic rhinitis, its role in allergic asthma appears more complex.

In a large randomized, double-blind, placebo-controlled trial (26), the addition of an HDM SLIT tablet to standard therapy in adults with inadequately controlled asthma significantly reduced the risk of moderate-to-severe exacerbations during a standardized inhaled corticosteroid step-down phase, with hazard ratios of 0.72 and 0.69 for the two active doses compared with placebo, corresponding to an absolute risk reduction of approximately 9%–10%. This pivotal trial demonstrated a reduction in asthma exacerbations, which was consistent across secondary endpoints related to symptom worsening and lung function decline, supporting a potentially beneficial effect on disease control. In contrast, no significant improvements were observed in asthma control or QoL scores, suggesting that SLIT may preferentially impact future risk, particularly exacerbations, rather than day-to-day symptom control in patients with allergic asthma.

Additional evidence on the effectiveness of SLIT in allergic asthma was derived from a prospective, randomized, controlled study (27) evaluating three years of HDM SLIT in 332 patients with HDM-induced nasobronchial allergies. In this study, SLIT alone or in combination with pharmacotherapy was associated with a significant and progressive reduction in both rhinitis and asthma symptom scores over time compared with pharmacotherapy alone. Asthma control also improved significantly, as reflected by increased Asthma Control Test (ACT) scores and a reduction in medication use. Notably, SLIT demonstrated a steroid-sparing effect, with a significant reduction in both the dose and duration of inhaled corticosteroid use over the three-year treatment period, whereas no significant changes were observed in the control group. Overall, these findings support the role of SLIT as an effective adjunctive therapy in patients with HDM-driven allergic asthma, contributing not only to symptom control but also to a reduction in the pharmacological treatment burden.

These findings are further strengthened by long-term real-world evidence from the REACT study (28), a large retrospective propensity score-matched cohort including over 46,000 patients treated with allergen immunotherapy (AIT), comprising both SCIT and SLIT, with allergic rhinitis, followed for up to nine years. In patients with pre-existing asthma, AIT was associated with sustained reductions in both controller and reliever medication use, a higher likelihood of stepping down asthma treatment, and a significantly lower risk of severe asthma exacerbation over time. Notably, these effects were consistent across multiple clinically relevant outcomes and were maintained throughout long-term follow-up, supporting durable improvement in asthma control under real-world conditions. The REACT study did not provide SLIT-specific analyses, and its findings should be interpreted in the context of a broader body of evidence. However, because of its exceptionally large sample size and long follow-up period, it remains one of the most informative real-world studies available on the long-term effectiveness of AIT. Taken together, these data reinforce the evidence from RCTs and suggest that SLIT, as part of allergen immunotherapy, may contribute not only to improving asthma control but also to reducing exacerbation risk and limiting disease progression over time.

### Pediatric vs. adult populations

Compared with adults, evidence supporting SLIT in pediatric populations has historically been more limited, mainly due to the later development of dedicated pediatric RCTs and the smaller sample sizes of early studies. Nevertheless, in recent years, an increasing number of studies have provided more robust evidence supporting the efficacy and safety of SLIT in children with respiratory allergic diseases. In this context, a large phase III randomized, double-blind, placebo-controlled trial (29) evaluated the efficacy and safety of an HDM SLIT tablet in 1,460 children aged 5–11 years with HDM-induced allergic rhinitis with or without asthma. Treatment with SLIT resulted in a significant reduction in the total combined rhinitis score compared with placebo, corresponding to a relative reduction of 22% (*p* < 0.0001), along with significant improvements in symptom scores, medication use, and disease-related QoL. Clinical improvement was observed as early as eight weeks after treatment initiation. Asthma-related outcomes also favored SLIT, with reductions in symptom scores and nocturnal awakenings, although the study was not specifically powered to evaluate asthma efficacy. The safety profile was favorable, with adverse events predominantly mild local reactions and very low discontinuation rates due to treatment-related adverse events (2.5%).

Further evidence supporting the efficacy of SLIT in pediatric populations was derived from a *post hoc* analysis of two Japanese phase III randomized controlled trials (30) evaluating the 300 IR HDM SLIT-tablet in children and adolescents with moderate-to-severe HDM allergic rhinitis. In the overall pediatric population, treatment significantly improved both symptom and combined symptom-medication scores compared with placebo, with relative reductions of approximately 13%. Notably, treatment effects were substantially greater in patients with the highest disease activity, reaching reductions of up to 27%–34% in symptom and combined scores across the pediatric and adolescent subgroups.

Evidence supporting the efficacy of SLIT in pediatric populations has also been extended to seasonal allergens. In a large phase III randomized, double-blind, placebo-controlled trial (31) including 952 children and adolescents (5–17 years) with moderate-to-severe tree pollen-induced allergic rhinoconjunctivitis, treatment with the SQ tree SLIT-tablet resulted in a significant and clinically relevant reduction in the combined symptom-medication score during the birch pollen season compared with placebo (−21.9%; *p* = 0.0004), together with significant improvements in both symptom and medication scores. Clinical benefits were consistently observed throughout the entire tree pollen season, accompanied by improvements in QoL and symptom-free days. The treatment was generally well tolerated, with most adverse events consisting of mild local reactions.

Overall, these findings further support the efficacy and safety of SLIT across pediatric age groups and suggest that treatment effects in children are broadly comparable to those observed in adult populations for perennial and seasonal respiratory allergies.

However, not all pediatric RCTs have demonstrated statistically significant efficacy. In a recent phase III trial (32) involving 533 children and adolescents with HDM-driven allergic asthma, SLIT did not significantly reduce clinically relevant asthma exacerbations compared with placebo. The interpretation of these findings is limited by the substantial reduction in exacerbation rates during the COVID-19 pandemic, which likely reduced the statistical power. Nevertheless, the exacerbation rates numerically favored active treatment, whereas safety and immunological findings remained consistent with a biologically active intervention. Similarly, in an RCT (33) with 111 children with HDM-induced mild-to-moderate asthma, SLIT did not improve asthma symptoms, medication use, lung function, or rhinitis outcomes beyond optimized conventional management. However, it significantly increased allergen-specific IgG4 and IgE levels and reduced skin test reactivity, confirming its immunological activity, despite limited additional clinical benefits.

Overall, RCTs consistently support the efficacy of SLIT in allergic rhinitis across different allergens and age groups, particularly in reducing symptoms and medication use (Table 1). Evidence in allergic asthma remains heterogeneous, although several studies suggest beneficial effects on exacerbation risk and treatment burden. Long-term observational data further support the potential disease-modifying role of SLIT.

**Table 1:** Summary of randomized controlled trials and real-world studies evaluating SLIT in respiratory allergic diseases discussed in this review.

Study	Design	Population	SLIT product	Treatment/Follow-up	Main outcomes
Durham et al. (19)	Randomized, double-blind, phase III, placebo-controlled trial	634 (18-65 years old)	Grass pollen (Grazax)	3 season of treatment/ + 2 years of f-up	DSS reduced by 25–36%; DMS reduced by 20–45%; RCS reduced by 27–41%.
Devillier et al. (20)	Real-World Analysis	1,099 SLIT patients vs. 27,475 controls (> 5 years old)	Grass pollen (Oralair or Grazax)	≥ 2 season of treatment/ + 2 years of f-up	50% decrease of symptomatic AR medication dispensing; 63.7% lower risk of new asthma onset after age matching (95% CI 31.5–80.7%).
Zielen et al. (21)	Real-World analysis	2,851 SLIT patients vs. 71,275 controls (> 5 years old)	Grass pollen (Oralair or Grazax)	≥ 2 season of treatment/ + 2 years of f-up	Lower AR medication use after SLIT cessation (−18.8% vs. controls; *P* < .001); lower risk of asthma onset (OR 0.696; *P* = .002) and longer time to asthma onset (HR 0.523; *P* = .003); lower asthma medication after SLIT cessation (−16.7% vs. controls; *P* = .004).
Demoly et al. (22)	Real-World analysis (on EfficAPSI cohort)	112,492 SLIT patients vs. 333,082 controls (no age limits)	Multiple allergens	≥ 1 year of treatment/ + 2 years of f-up	Lower risk of asthma onset vs. control [HR (95% CI) = 0.62]; 33.3% reduction in GINA step-up regardless baseline GINA steps.
Wahn et al. (23)	Real-World analysis	14,018 AIT patients (1,282 SLIT patients) vs. 433.140 controls (≥ 5 years old)	Birch pollen	≥ 2 season of treatment/ + 2 years of f-up	More SLIT (74.4%) vs. non-AIT (47.4%) patients were AR medication-free on f-up [OR (95% CI): 0.31; *P* < 0.001]; more SLIT (53.9%) vs. non-AIT (35.1%) patients were asthma medication-free on f-up [OR (95% CI): 0.59; *P* < 0.001]; reduced new-onset asthma risk in the SLIT vs. non-AIT group (OR: 0.66; *P* = 0.013) during treatment.
Okamoto et al. (24)	Randomized, double-blind, phase III, placebo-controlled trial	968 patients (≥ 12 years old) randomized 1: 1: 1 to 300 IR, 500 IR, or placebo	*D. pteronyssinus* and *D. farinae* (1: 1 mixture)	1 year of treatment/no f-up	AASS in the last 8 weeks of treatment significantly improved in both 300 IR and 500 IR groups compared to that in the placebo group (*P* < 0.001).
Okamoto et al. (25)	Real-World analysis	538 patients	300 IR HDM	Up to 4 years of treatment/ ≥ 1 years of f-up	Severe AR decreased from 46.4% to 1.0% and asymptomatic patients increased from 0.3% to 57.7% at 4 years; AR medication use decreased from 66.0% to 43.3%.
Virchow et al. (26)	Randomized, double-blind, placebo-controlled trial	834 asthmatic patients (≥ 18 years old), randomized 1:1:1 to placebo or 6 SW-HDM or 12 SQ-HDM SLIT tablets	*D. pteronyssinus* and *D. farinae*	6–12 months of treatment	Reduced moderate-to-severe asthma exacerbation risk vs. placebo [HR 0.72, 95% CI 0.52–0.99 [6 SQ-HDM]; HR 0.69, 95% CI 0.50–0.96 [12 SQ-HDM]]; absolute risk reduction: 9–10%. No significant improvement in asthma control or QoL.
Baba et al. (27)	Prospective, randomized, parallel-group controlled study	332 patients	2,800 Biological Units (BU) of HDM (*D. farinae*, *D. pteronyssus*, and *Blomia*)	3 years of treatment/no further f-up	Improved rhinitis and asthma symptoms and reduced medication; ICS dose decreased from 340 ± 90 to 210 ± 58 μg/day in the SLIT group at 3 years (*P* = .001); reduced neosensitization.
Fritzsching et al. (REACT) (28)	Retrospective, real-world cohort study	46,024 AIT treated patients (8,570 SLIT), matching 1: 1 with controls	Multiple allergens (both seasonal and perennial)	Mean of 18 months of treatment/up to 9 years of f-up	Sustained reductions in AR and asthma medication use; greater likelihood of asthma treatment step-down (*P* < .0001); greater reduction in severe asthma exacerbations (*P* < .05).
Schuster et al. (29)	Randomized, double-blind, phase III, placebo-controlled trial	729 SLIT patients (5–11 years old) vs. 731 controls	standardized allergen extract from *D. pteronyssinus* and *D. farinae*	1 year of treatment/no further f-up	TCRS reduction of 22.0% in SLIT group (95% CI: 12.0, 31.1); with improvements in symptom, medication scores and QoL. Asthma symptoms and medication use were also reduced.
Devillier et al. (30)	Post hoc analysis of two phase III randomized controlled trials	438 pediatric patients (5–16 years) from the JP-D1732 study and a pooled cohort of 270 adolescents (12–17 years) from both studies (JP-D1731 and JP-D1732).	*D. pteronyssinus* and *D. farinae* (1: 1 mixture)	1 year of treatment	Reduced AASS and ACSMS vs. placebo (−13.1% and −12.9% in patients aged 5–16 years; −19.9% and −21.2% in adolescents), with greater benefits in patients with the highest baseline symptom burden (up to −32.5% and −34.1%, respectively).
Gappa et al. (31)	Randomized, double-blind, phase III, placebo-controlled trial	952 children (5–17 years old) randomized 1: 1 to SLIT or placebo	12 SQ tree SLIT-tablet with standardized Birch (*Betula verrucosa*) pollen extract	1 year of treatment	Significant improvement in TCS vs. placebo (absolute difference: 1.29, 95% CI 0.58–2.00; *P* = .0004), corresponding to a 21.9% relative reduction (95% CI 10.6–31.9). Improvements were also observed in symptom and medication scores.
Roberts et al. (32)	Randomized, double-blind, phase III, placebo-controlled trial	533 children (5–17 years old) randomized 1: 1 to SLIT or placebo	12 SQ HDM SLIT-tablet containing standardized extract from *D. pteronyssinus* and *D. farinae*	24–30 months	No significant reduction in clinically relevant asthma exacerbations vs. placebo (rate ratio 0.89, 95% CI 0.60–1.31), possibly influenced by low exacerbation rates during the COVID-19 pandemic.
Pham-Thi et al. (33)	Randomized, double-blind, placebo-controlled trial	111 children (5–17 years old) randomized 1: 1 to SLIT or placebo	HDM extract SLIT tablets	18 months	No significant benefit vs. placebo in asthma symptoms, medication use, lung function, or rhinitis symptoms despite significant immunologic effects (reduced HDM skin reactivity, *P* < 0.01; increased HDM-specific IgE and IgG4, *P* < 0.001).

RCT, Randomized Controlled Trial; DSS, Daily Symptom Score; DMS, Daily Medication Score; RCS, Rhinoconjunctivitis Combined Score; AR, Allergic Rhinitis; IR, Index of Reactivity; AASS, Average Adjusted Symptom Score; HDM, House Dust Mite; ICS, inhaled corticosteroid; TCRS, Total Combined Rhinitis Score; ACSMS, Average Combined Symptom and Medication Score.

## Evidence from meta-analyses

One of the largest EAACI systematic reviews and meta-analyses (34), including 98 studies and more than 7,000 patients with allergic asthma, reported significant reductions in both asthma symptoms and medication scores following AIT (SMD −1.11 and −1.21, respectively). However, the analysis also revealed substantial between-study heterogeneity and possible publication bias, mainly related to the predominance of small studies with highly favorable outcomes. Interestingly, indirect comparisons suggested greater efficacy of SCIT than that of SLIT in asthma, although both approaches maintained an overall favorable safety profile.

Similarly, meta-analyses focusing on seasonal allergic rhinitis have confirmed the efficacy of SLIT. A large analysis including 19 double-blind placebo-controlled trials (35) involving nearly 3,000 patients demonstrated significant reductions in both symptom and medication scores with grass pollen SLIT, although the overall clinical benefit was considered modest (SMD, −0.32 and −0.33, respectively). Importantly, efficacy appeared greater in adults than in children and was associated with higher allergen doses and longer pre-seasonal treatment schedules, suggesting a relevant dose-response relationship and highlighting the importance of treatment optimization. However, substantial heterogeneity persisted across studies, likely reflecting differences in the allergen formulations, dosing regimens, patient populations, and study methodologies.

Recent evidence has progressively shifted from evaluating AIT as a homogeneous therapeutic class toward product- and allergen-specific analyses. A systematic review and meta-analysis (36) focusing exclusively on house dust mite (HDM) SLIT tablets demonstrated significant improvements in combined symptom and medication scores (SMD −0.28), rhinitis symptom scores (SMD −0.27), and QoL outcomes, with consistently low heterogeneity across most analyses. Importantly, efficacy was observed across different HDM tablet formulations, doses, and age groups, supporting the robustness and reproducibility of the standardized HDM-SLIT tablet protocols. The low degree of variability observed in this analysis contrasts with the substantial heterogeneity reported in earlier pooled AIT meta-analyses and further supports the concept that product-specific analyses may provide more reliable estimates of efficacy than conventional pooled analyses combining heterogeneous allergen extracts and treatment protocols.

Similarly, a recent meta-analysis (37) specifically evaluating a five-grass pollen liquid SLIT formulation confirmed significant reductions in symptom and medication scores compared with placebo, with effect sizes comparable to those reported for SLIT tablet use. Notably, heterogeneity markedly decreased after the exclusion of a limited number of outlier studies, further emphasizing the importance of carefully standardizing and using product-specific approaches in modern AIT research.

Importantly, the strength and consistency of the evidence varied considerably according to the allergen source. In contrast to the more robust evidence available for pollen or HDM allergy, the evidence for mold allergy appeared less consistent, with a systematic review (38) reporting low overall quality of evidence, substantial heterogeneity, and small study populations, with more favorable results for *Alternaria* than for *Cladosporium* extracts.

Nevertheless, a GRADE-based systematic review (39) on childhood asthma questioned the applicability of earlier AIT evidence to current clinical practice, emphasizing that many historical trials were conducted before the widespread use of inhaled corticosteroids and standardized asthma management, resulting in substantial indirectness, very low overall quality of evidence, and limited availability of standardized patient-relevant outcomes.

Overall, current meta-analytic evidence supports the efficacy and safety of AIT in respiratory allergic diseases, while also emphasizing the need for more standardized, product-specific, and methodologically rigorous studies to better define long-term outcomes and optimal patient selection (Table 2).

**Table 2:** Summary of systematic reviews and meta-analyses evaluating SLIT in respiratory allergic diseases discussed in this review.

Study	Design	Intervention/allergen	Main findings	Main limitations
Dhami et al. (34)	SR and MA (including 98 studies)	AIT (SCIT and SLIT); multiple allergens and formulations	Reduced short-term symptom scores (SMD −1.11, 95% CI −1.66 to −0.56) and medication scores (SMD −1.21, 95% CI −1.87 to −0.54). No significant reduction in short-term combined symptom-medication scores (SMD 0.17, 95% CI −0.23 to 0.58).	Heterogeneity across studies; evidence of possible publication bias; heterogeneous AIT routes/products; limited evidence for long-term outcomes.
Di Bona et al. (35)	SR and MA (including 19 RCTs)	Grass pollen SLIT vs. placebo	SLIT significantly reduces symptoms score (SMD, −0.32; 95% CI, −0.44 to −0.21) and medication use (SMD, −0.33; 95% CI, −0.50 to −0.16) vs. placebo; greater efficacy was observed in adults than in children and with treatment duration of ≥ 12 weeks.	Heterogeneity across studies in populations, SLIT regimens/doses and treatment duration; overall clinical benefit was modest.
Kulalert et al. (36)	SR and MA (including 8 RCTs)	HDM SLIT tablets vs. placebo	Reduced CSMS vs. placebo (SMD −0.28, 95% CI −0.32 to −0.23), with significant improvements in rhinitis symptom and medication scores, conjunctivitis symptoms, and QoL.	Heterogeneous scoring systems and study characteristics; studies conducted in Japan, Europe, and North America, limiting generalizability to other populations.
Di Bona et al. (37)	SR and MA (including 8 RCTs)	5-grass pollen liquid SLIT vs. placebo	Reduced SS (SMD −0.34, 95% CI −0.62 to −0.06) and MS (SMD −0.54, 95% CI −0.97 to −0.10) vs. placebo; no significant differences on age or sensitization status.	Small sample size of available RCTs; heterogeneity across study populations and treatment protocols.
Di Bona et al. (38)	SR (including 9 RCTs)	Mainly *Alternaria* and *Cladosporium* extracts (only 2 RCTs on SLIT)	Low-strength evidence in reductions of symptoms and medication use, although only 4/9 studies favored AIT over comparators; greater effects were reported in studies with longer-f-up (MS: SMD −3.96 to −3.97).	Small number and sample size of studies; inconsistent results; heterogeneous AIT routes/products; overall low strength of evidence.
van de Griendt et al. (39)	GRADE-based SR (including previous SRs and RCTs)	AIT (SCIT and SLIT); multiple aeroallergens in children with allergic asthma	SLIT: very low-quality evidence, with clinical heterogeneity and non-standardized symptom scores preventing quantitative synthesis of asthma symptom outcomes. Overall, evidence was insufficient to determine the added effectiveness of AIT in children receiving contemporary asthma treatment with ICS.	Serious risk of bias, large clinical heterogeneity between studies and lack of applicability because studies were performed in the pre-ICS era

SR, Systematic Review; RCT, Randomized Controlled Trial; SMD, Standard Mean Difference; CSMS, combined symptom and medication score.

## Limitations of the evidence

Despite the overall positive evidence, several limitations should be acknowledged when interpreting the efficacy of SLIT. First, substantial heterogeneity across randomized controlled trials remains a major concern. Differences in study populations (age, disease severity, mono- vs. polysensitization), allergen sources (pollens, house dust mites, molds), and outcome definitions contribute to variability in reported treatment effects and limit direct comparability across studies. Second, dosing regimens and treatment schedules are highly variable, ranging from pre-seasonal to continuous administration and involving different allergen concentrations and formulations (tablets vs. drops). This variability complicates the identification of optimal dosing strategies and may partly explain the inconsistencies in efficacy, particularly in meta-analytic estimates. Third, methodological limitations affect some of the available evidence. Several trials have relatively small sample sizes, short follow-up durations, or incomplete reporting of clinically relevant outcomes, such as long-term disease modification. In addition, the risk of bias cannot be excluded in some studies due to issues related to randomization procedures, blinding, or selective outcome reporting. Similarly, real-world studies complement RCT evidence by reflecting routine clinical practice; however, their findings should be interpreted with caution because they remain susceptible to residual confounding and treatment-selection bias. In particular, these studies may be affected by confounding by indication, whereby treatment is preferentially prescribed to patients with specific disease characteristics or prognostic profiles; healthy-adherer bias, whereby patients who initiate and persist with SLIT may also engage more consistently in other health-promoting behaviors; outcome misclassification when asthma onset is inferred from prescription or medication-dispensing proxies rather than confirmed clinical diagnoses; and potential sponsorship bias, given that several key cohorts were supported by industry. Publication bias may further inflate the perceived efficacy, as studies with positive findings are more likely to be published. Finally, the frequent pooling of heterogeneous AIT products in earlier meta-analyses may have obscured product-specific differences, reducing the clinical applicability of the aggregated results. Taken together, these factors highlight the need for more standardized, adequately powered, and methodologically rigorous trials focusing on well-defined SLIT formulations and clinically meaningful outcomes to confirm these findings.

## Clinical implications and future perspectives

SLIT is now firmly established in clinical practice as a potential first-line disease-modifying treatment for patients with IgE-mediated allergic rhinitis, with or without concomitant asthma, particularly when symptoms are inadequately controlled by pharmacotherapy or when patient preference favors avoiding injections (6). Its primary indication extends to individuals seeking long-term symptom control, given SLIT's ability to induce sustained clinical benefits after treatment discontinuation when administered for an adequate duration. A critical determinant of SLIT success is patient adherence; since the treatment is self-administered at home, adherence relies heavily on patient motivation, clear education, and regular follow-ups (40). Real-world evidence confirms that while adherence can be challenging, structured support strategies significantly improve persistence and clinical outcomes, with long-term persistence being essential to achieve the recommended minimum three-year course for durable immunity (12). From a practical perspective, treatment feasibility is supported by a favorable safety profile, with adverse events predominantly consisting of mild-to-moderate local reactions, whereas severe systemic reactions are rare. However, appropriate patient selection remains essential, particularly in patients with uncontrolled asthma, and treatment initiation should follow product-specific recommendations, including supervised first administration where required (10).

Future developments are increasingly focused on overcoming these historical limitations through product standardization and novel delivery formats. The advent of standardized SLIT tablets (e.g., for grass pollen, house dust mites, and tree pollen) has markedly improved reproducibility, dosing precision, and safety compared to earlier drop formulations, facilitating their wider adoption in routine care (13). Furthermore, ongoing research is exploring optimized dosing regimens, including accelerated up-dosing schedules, and the potential of combining SLIT with adjuvants to enhance immune tolerance. Despite the robust evidence base, further high-quality research is needed to refine patient selection criteria, particularly for complex phenotypes such as polysensitized patients or those with severe asthma, and to better define the long-term disease-modifying effects across diverse allergen sources.

The key clinical message is that SLIT should be considered early in the management algorithm for appropriate candidates, as it offers the unique potential to alter the natural history of respiratory allergy, reduce medication burden, and potentially influence the long-term course of the disease, provided that treatment is standardized, adequately dosed, and consistently maintained.
